# Implementing rapid testing for tuberculosis in Mozambique

**DOI:** 10.2471/BLT.14.138560

**Published:** 2014-11-26

**Authors:** James Cowan, Cathy Michel, Ivan Manhiça, Claudio Monivo, Desiderio Saize, Jacob Creswell, Stephen Gloyd, Mark Micek

**Affiliations:** a Health Alliance International, 1107 NE 45th Street, Suite 350, Seattle, 98105, United States of America.; b Mozambican National Tuberculosis Control Programme, Maputo, Mozambique.; c Stop TB Partnership, World Health Organization, Geneva, Switzerland.

## Abstract

**Problem:**

In Mozambique, pulmonary tuberculosis is primarily diagnosed with sputum smear microscopy. However this method has low sensitivity, especially in people infected with human immunodeficiency virus (HIV). Patients are seldom tested for drug-resistant tuberculosis.

**Approach:**

The national tuberculosis programme and Health Alliance International introduced rapid testing of smear-negative sputum samples. Samples were tested using a polymerase-chain-reaction-based assay that detects *Mycobacterium tuberculosis* deoxyribonucleic acid and a mutation indicating rifampicin resistance; Xpert® MTB/RIF (Xpert®). Four machines were deployed in four public hospitals along with a sputum transportation system to transfer samples from selected health centres. Laboratory technicians were trained to operate the machines and clinicians taught to interpret the results.

**Local setting:**

In 2012, Mozambique had an estimated 140 000 new tuberculosis cases, only 34% of which were diagnosed and treated. Of tuberculosis patients, 58% are HIV-infected.

**Relevant changes:**

From 2012–2013, 1558 people were newly diagnosed with tuberculosis using sputum smears at intervention sites. Xpert® detected *M. tuberculosis* in an additional 1081 sputum smear-negative individuals, an increase of 69%. Rifampicin resistance was detected in 58/1081 (5%) of the samples. However, treatment was started in only 82% of patients diagnosed by microscopy and 67% of patients diagnosed with the rapid test. Twelve of 16 Xpert® modules failed calibration within 15 months of implementation.

**Lessons learnt:**

Using rapid tests to diagnose tuberculosis is promising but logistically challenging. More affordable and durable platforms are needed. All patients diagnosed with tuberculosis need to start and complete treatment, including those who have drug resistant strains.

## Résumé

**Problème:**

Au Mozambique, la tuberculose pulmonaire est principalement diagnostiquée en examinant des frottis d'expectoration au microscope. Cependant, cette méthode est peu sensible, en particulier chez les personnes infectées par le virus de l'immunodéficience humaine (VIH). Les patients sont rarement testés pour la tuberculose résistante aux médicaments.

**Approche:**

Le programme national de lutte contre la tuberculose et Health Alliance International ont introduit le dépistage rapide sur les échantillons de frottis d'expectoration négatifs. Les échantillons ont été testés à l’aide d’un test basé sur l'amplification en chaîne par polymérase, qui détecte l'acide désoxyribonucléique de la bactérie *Mycobacterium tuberculosis* et une mutation indiquant la résistance à la rifampicine: Xpert® MTB/RIF (Xpert®). Quatre machines ont été déployées dans quatre hôpitaux publics avec un système de transport des frottis d'expectoration pour transférer les échantillons depuis les centres de santé sélectionnés. Les techniciens de laboratoire ont été formés à utiliser les machines et les cliniciens ont appris à interpréter les résultats.

**Environnement local:**

En 2012, le nombre de nouveaux cas de tuberculose au Mozambique était estimé à 140 000, dont seuls 34% étaient diagnostiqués et traités. Des patients atteints de tuberculose, 58% étaient infectés par le VIH.

**Changements significatifs:**

Depuis 2012–2013, 1558 personnes ont été nouvellement diagnostiquées comme tuberculeuses par examen des frottis d'expectoration dans les sites d'intervention. Xpert® a détecté *M. tuberculosis* dans 1081 échantillons supplémentaires, soit une augmentation de 69%. La résistance à la rifampicine a été détectée dans 58 des 1 081 échantillons (5%). Cependant, le traitement avait commencé chez seulement 82% des patients diagnostiqués par microscopie et 67% des patients diagnostiqués par le test de dépistage rapide. Parmi les 16 modules Xpert®, 12 d'entre eux n'avaient pas réussi l'étalonnage au cours des 15 mois de la mise en œuvre.

**Leçons tirées:**

L'utilisation de tests de dépistage rapide pour diagnostiquer la tuberculose est prometteuse, mais reste difficile sur le plan logistique. Des plateformes plus abordables et durables sont nécessaires. Tous les patients diagnostiqués comme tuberculeux doivent commencer et terminer leur traitement, y compris ceux avec des souches résistantes aux médicaments.

## Resumen

**Situación:**

En Mozambique, la tuberculosis pulmonar se diagnostica principalmente mediante la baciloscopia del esputo. No obstante, la sensibilidad de este método es insuficiente, especialmente en las personas infectadas con el virus de la inmunodeficiencia humana (VIH). Pocas veces se somete a los pacientes a pruebas para la tuberculosis resistente a medicamentos.

**Enfoque:**

El programa nacional contra la tuberculosis y Health Alliance International han introducido la prueba rápida de muestras de esputo de frotis negativo. Se analizaron las muestras mediante un análisis basado en la reacción en cadena de la polimerasa que detecta el ácido desoxirribonucleico de la *Mycobacterium tuberculosis* y una mutación que indica resistencia a la rifampicina; Xpert® MTB/RIF (Xpert®). Se instalaron cuatro máquinas en cuatro hospitales públicos junto con un sistema de transporte de esputo para transferir las muestras de los centros de salud seleccionados. Se capacitó a los técnicos de laboratorio para operar las máquinas y a los médicos se les enseñó a interpretar los resultados.

**Marco regional:**

En 2012, Mozambique presentó aproximadamente 140 000 casos nuevos de tuberculosis, de los cuales solo se diagnosticó y trató el 34 %. De los enfermos de tuberculosis, 58% está infectado por el VIH.

**Cambios importantes:**

Entre 2012 y 2013, se diagnosticaron 1558 afectados nuevos por tuberculosis por medio del frotis de esputo en los centros de intervención. Xpert® detectó la *M. tuberculosis* en 1081 muestras adicionales, lo que representa un aumento del 69 %. Se detectó resistencia a la rifampicina en 58 de las 1081 muestras (5 %). Sin embargo, el tratamiento solo se inició en el 82 % de los pacientes diagnosticados por baciloscopia y en el 67 % de los pacientes diagnosticados mediante la prueba rápida. Hubo fallos de calibración en doce de los 16 módulos de Xpert® a los 15 meses de la aplicación.

**Lecciones aprendidas:**

El uso de pruebas rápidas para diagnosticar la tuberculosis resulta prometedor, pero es difícil desde el punto de vista. Se requieren plataformas más asequibles y duraderas. Todos los pacientes diagnosticados con tuberculosis deben iniciar y completar un tratamiento, incluidos aquellos con cepas resistentes a medicamentos.

## ملخص

**المشكلة:**

يتم تشخيص السل الرئوي، في موزامبيق، بشكل رئيسي عن طريق الفحص المجهري باستخدام لطاخة البلغم. ولكن هذا الأسلوب يتمتع بحساسية منخفضة، لا سيما لدى الأشخاص المصابين بفيروس العوز المناعي البشري. ونادراً ما يتم فحص المرضى لتحديد الإصابة بالسل المقاوم للأدوية.

**الأسلوب:**

قام البرنامج الوطني لمكافحة السل والتحالف الدولي للصحة بتنفيذ الاختبار السريع لعينات البلغم الإيجابية للطاخة. وتم اختبار العينات باستخدام اختبار يستند على تفاعل البوليميراز المتسلسل الذي يكشف البكتريا المتفطرة السلية والحمض الريبي النووي منزوع الأكسجين ومقاومة الريفامبيسين التي تشير إلى حدوث طفرة؛ واختبار داء السل "Xpert® MTB/RIF" (Xpert®). وتم نشر أربعة أجهزة في أربعة مستشفيات عمومية مع نظام نقل البلغم لنقل العينات من المراكز الصحية المختارة. وتم تدريب فنيي المختبرات على تشغيل الأجهزة وتثقيف الخبراء السريريين على تفسير النتائج.

**المواقع المحلية:**

في عام 2012، بلغت حالات السل الجديدة في موزامبيق وفق التقديرات 140000 حالة، ولم يتم تشخيص وعلاج سوى 34 % منها. وكانت نسبة ستين في المائة من مرضى السل مصابة بفيروس العوز المناعي البشري.

**التغيّرات ذات الصلة:**

من عام 2012 إلى عام 2013 تم تشخيص إصابة 1558 شخصاً حديثاً بالسل باستخدام لطاخات البلغم في مواقع التدخل. وكشف اختبار Xpert® البكتريا المتفطرة السلية في 1081 عينة إضافية، بزيادة نسبتها 69 %. وتم كشف مقاومة الريفامبيسين في 58/1081 (5 %) من العينة. ومع ذلك، تم بدء العلاج في 82 % فقط من المرضى الذين تم تشخيصهم عن طريق الفحص المجهري و67 % من المرضى الذين تم تشخيصهم باستخدام الاختبار السريع. وعجزت اثني عشرة وحدة من أصل 16 وحدة لاختبارات Xpert® عن المعايرة في غضون 15 شهراً من التنفيذ.

**الدروس المستفادة:**

استخدام الاختبارات السريعة في تشخيص السل أمر واعد ولكنه ينطوي على تحديات من الناحية اللوجستية. وتوجد ثمة حاجة إلى منصات أكثر ذات تكلفة معقولة وتتسم بالقوة. ويحتاج جميع المرضى الذين يتم تشخيص إصابتهم بالسل إلى بدء العلاج وإتمامه، بما في ذلك المرضى المصابين بسلالات مقاومة للأدوية.

## 摘要

**问题:**

在莫桑比克，肺结核主要使用痰涂片镜检确诊。然而，这种方法灵敏度较低，特别是在病人感染了艾滋病病毒（HIV）的时候。患者很少接受耐药结核病检测。

**方法:**

国家结核病项目和国际健康联盟推出使用涂片阴性痰标本的快速检测。样品使用以聚合酶链反应为基础的化验进行检测，该化验为Xpert®MTB/RIF（Xpert®），可发现分枝杆菌肺结核脱氧核糖核酸和指示利福平抗性的突变。在四所公立医院部署了四台机器和痰运输系统，以从选定的医疗中心转送样本。培训实验室技术人员来操作这些机器并指导临床医生解释结果。

**当地状况:**

2012年，莫桑比克估计有14万名新结核病例，其中只有34%接受诊断和治疗。58%的结核病患者感染艾滋病毒。

**相关变化:**

从2012至2013年，在干预点通过痰涂片诊断1558例新确诊结核病患者。Xpert®在额外1081个样本中发现结核分枝杆菌，增加达69%。在这1081例样本中有58例（5%）发现利福平抗性。然而，显微镜诊断的患者中只有82%开始接受治疗，快速检测诊断的患者中只有67%开始接受治疗。在实施的16个月中，15个Xpert®模块中有12个没有校准。

**经验教训:**

使用快速检测来诊断肺结核是有希望的，但在逻辑上具有挑战性。需要更多实惠且耐用的平台。所有确诊患有肺结核的患者需要开始接受并完成治疗，包括那些带有耐药菌株的患者。

## Резюме

**Проблема:**

Диагностика туберкулеза легких в Мозамбике в основном проводится методом микроскопии мазка мокроты. Однако этот метод имеет низкую чувствительность, особенно у людей, инфицированных вирусом иммунодефицита человека (ВИЧ). Пациенты редко проверяются на наличие лекарственно-устойчивого туберкулеза.

**Подход:**

Национальная программа борьбы с туберкулезом и некоммерческая организация Health Alliance International внедрили метод экспресс-тестирования мазков мокроты с отрицательным результатом при микроскопии. Образцы мокроты проверялись путем анализа на основе полимеразной цепной реакции, который определяет наличие дезоксирибонуклеиновой кислоты *Mycobacterium tuberculosis* и мутации, указывающие на устойчивость к рифампицину. Данный экспресс-тест называется Xpert® MTB/RIF (Xpert®). В четырех государственных больницах были развернуты четыре установки и организована транспортная система для передачи образцов мокроты из избранных медицинских центров. Было проведено обучение лаборантов работе на данных установках и обучение врачей методу интерпретации результатов.

**Местные условия:**

Согласно оценкам, в 2012 году в Мозамбике возникло примерно 140 000 новых случаев туберкулеза, лишь 34% из которых были диагностированы и вылечены. При этом 58% больных туберкулезом являются одновременно ВИЧ-инфицированными.

**Осуществленные перемены:**

За период 2012-2013 гг. туберкулез был диагностирован у 1558 человек по результатам анализов мазков мокроты, проведенных в медицинских учреждениях. При анализе по методу Xpert® были обнаружены микобактерии в дополнительных 1081 образцах, что составляет увеличение на 69%. Устойчивость к рифампицину была обнаружена в 58 из 1081 (5%) образцов. Однако лечение было начато только в отношении 82% пациентов с диагнозом, полученным методом микроскопии, и 67% пациентов с диагнозом, полученным методом экспресс-тестирования. Кроме того, 12 из 16 модулей Xpert® не прошли калибровку в течение 15 месяцев после начала использования.

**Выводы:**

Использование экспресс-тестов для диагностики туберкулеза является перспективным методом, который сложно реализовать с логистической точки зрения. Необходимы более доступные и надежные платформы. Все пациенты с диагнозом туберкулеза, в том числе те, у кого обнаружены резистентные штаммы, должны начать и завершить лечение**.**

## Introduction

People infected with human immunodeficiency virus (HIV) are susceptible to tuberculosis. This has led to an HIV-driven tuberculosis epidemic in sub-Saharan Africa over the past two decades.^1^ Diagnosing tuberculosis is often difficult given the low sensitivity of smear microscopy. Among people infected with HIV, smear microscopy is even less sensitive due to the lower bacterial load in co-infected individuals.^2^

Xpert® MTB/RIF (Xpert®; Cepheid, Sunnyvale, United States of America) is a rapid and fully-automated molecular test that uses real-time polymerase chain reaction on sputum samples to simultaneously detect *Mycobacterium tuberculosis* deoxyribonucleic acid and the genetic sequence indicative of rifampicin resistance.^3^^,^^4^ The test has 88% sensitivity for tuberculosis and 94% sensitivity for rifampicin resistance. Given the high sensitivity, the test is more effective than sputum smear microscopy in diagnosing tuberculosis in HIV-infected people. Therefore the World Health Organization (WHO) has recommended the use of Xpert® for diagnosis of tuberculosis in countries with high prevalence of HIV infection and multi-drug resistant tuberculosis (MDR-TB).^5^^,^^6^ The four-module Xpert® machine and desktop computer retails for 17 000 United States dollars (US$) in low-income countries and each cartridge (one per sample) costs an additional US$ 10.^7^ This paper describes the introduction of Xpert® in Mozambique.

## Local setting

Mozambique has a high burden of tuberculosis. In 2012, WHO estimated an incidence of 552 per 100 000 people, which corresponds to approximately 140 000 new cases. However, only 34% of these estimated cases are diagnosed and notified to the national tuberculosis programme. Most, (94%), new tuberculosis patients are tested for HIV^8^ and 58% are found to be co-infected. Tuberculosis diagnosis is done in people reporting a persistent cough, using smear microscopy on sputum samples.^8^ MDR-TB testing is seldom done since culture of *M. tuberculosis* is not routinely available.^8^ New cases typically receive first-line therapy. The treatment success rate is 85% for new sputum smear-positive patients who start treatment at a public treatment centre.^9^ First-line therapy typically includes isoniazid, rifampicin, pyrazinamide and ethambutol. Second-line therapy typically includes kanamycin, levofloxacin, ethionamide, cycloserine, ethambutol and pyrazinamide.

In 2012, an estimated 3.5% (4900 people) of the new estimated tuberculosis patients had MDR-TB; however, only 213 patients with MDR-TB started appropriate second-line treatment.^8^ Both in Mozambique and globally, the prevalence of MDR-TB tuberculosis is increasing. Therefore improved MDR-TB diagnostics and access to treatment are priorities for the Mozambican national tuberculosis programme and the global health community.^8^

## Planned intervention

To increase the number of people diagnosed and treated with tuberculosis and to improve MDR-TB detection and treatment, the Xpert® implementation project was approved by the Mozambican Ministry of Health, with support from Health Alliance International. We chose to use a two-step testing algorithm for individuals suspected of having pulmonary tuberculosis. All sputum samples were analysed using smear microscopy and new patients with at least one positive sputum smear out of two separate samples were treated with first-line tuberculosis therapy. Patients with two separate smear negative results were retested using Xpert® on one of the original samples.

In the beginning of February 2012, four Xpert® machines were deployed at two district and two urban public hospitals in four districts in Sofala and Manica provinces. We developed a transportation network to transfer smear negative sputum samples from three of the 10 surrounding health centres to the two urban hospitals. Eight remote health facilities routinely transferred samples to the two district hospitals via existing sputum transport networks. This covered approximately 5% of all sputum samples tested in Mozambique.^10^

Initial training and technical support for clinicians and laboratory staff in the four hospitals was led by Health Alliance International. Prior to Xpert® testing initiation, each site was given a day-long training session to train laboratory technicians to operate the machines and teach clinicians to interpret the results. Initial introductory meetings were also held with district and provincial ministry of health staff and supervisors. Health Alliance International trainers then provided at least weekly supervision visits to each site with focused training for technicians with elevated testing error rates, to troubleshoot problems, to review cartridge procurement and distribution and to undertake initial calibration. The trainers also monitored the testing care cascade to ensure that patients received their test results and were linked to appropriate treatment. Daily Xpert® testing and long-term management – such as technical support, cartridge procurement and routine maintenance – was assumed by the Ministry of Health after appropriate training.

The national tuberculosis programme registers new tuberculosis patients as either sputum smear negative or positive. The system has no capacity to record molecular test results. Therefore, laboratories using the rapid tests were instructed to report patients testing positive with Xpert® as sputum smear negative cases. However, this instruction was not always followed and an estimated 5–10% of sputum smear negative cases that tested positive on Xpert® were reported as sputum smear positive.

The performance of this project was monitored from reports generated from individual Xpert® machines. These reports were collected by hand each month, and information extracted included the number of tests run per quarter, a summary of the results, and lists of patients that tested positive, which were reconciled with the tuberculosis treatment registries. This was combined with quarterly data from the national tuberculosis programme on the reported number of sputum smears analysed and the number of patients started on tuberculosis treatment.

Xpert® was approved for general use by the Mozambican Ministry of Health and the national tuberculosis programme formally approved the implementation of this project. This implementation project was not a formal research study thus the University of Washington institutional review board declined to provide a full review of this proposal.

## Implementation and results

From 1 January 2012 to 31 December 2012; 1558/12 509 (12%) people tested at the four hospitals had sputum smear positive results. Of the 10 951 people with sputum smear negative results, 8631 (79%) were tested using Xpert® and 1081 (13%) were positive for tuberculosis. Thus, during this intervention Xpert® testing increased the diagnosis of bacteriologically-confirmed pulmonary tuberculosis by 1081/1558 (69%). Rifampicin resistance was detected in 58/1081 (5%) samples. Overall, 1019 (12%) of tests failed because of machine errors, and invalid or no results due to energy shortages.

We reviewed the records of 445 newly-diagnosed patients from the four hospitals in the second quarter of 2012 and found that 26% [115/445] had not been started on treatment (18% [35/200] of those diagnosed by microscopy and 33% [80/245] diagnosed using Xpert®). For patients with rifampicin resistance, 53% [31/58] were not started on second-line treatment. Three patients with rifampicin resistance were mistakenly initially started on first-line treatment but were converted to second-line treatment.

During 2012, 5076 people were placed on treatment in the four intervention districts, an increase of 632 patients (14.2%) over the baseline yearly average for these districts which was 4444 patients (15 554 patients were treated in these districts during the 3.5 years before implementation).

## Challenges

Despite encouraging outcomes, there were several programmatic and operational challenges in implementing and maintaining the Xpert® machines (Table 1).

**Table 1:** Responses to technical and logistical challenges encountered when implementing rapid tests for tuberculosis, Mozambique 2012–2013

Challenge	Reason	Response
High testing error rates	Laboratory technicians unfamiliar with Xpert®	Targeted training of technicians at facilities with high error rates. Error rate decreased from 12% in first year to 6% in the second year
	Higher (20%) cartridge error rates when cartridges were stored in non-air conditioned rooms	Installed air conditioners in cartridge storage areas
Loss to follow-up	Laboratory registries were not routinely reconciled with tuberculosis treatment registries	Developed monthly meetings to reconcile laboratory and treatment registries
	Current two-step testing algorithm for tuberculosis delays patient results, decreasing the percentage of patients started on tuberculosis treatment	Ensured that laboratories collect patient addresses and phone numbers
		Used existing community health workers to find and treat defaulters
		Start using Xpert® as a first-line test for high risk patients (those with HIV infection, diabetes, cancer, children) and those at risk for MDR-TB
Loss to follow-up for patients with rifampicin resistance	Some technicians did not initially understand that Xpert® tested for tuberculosis and rifampicin resistance	Additional staff education
		Pilot a remote monitoring system to immediately notify key individuals by SMS when a patient tests positive for rifampicin resistance
Lack of real-time monitoring and evaluation	Xpert® originally designed to transmit data via Ethernet cables, not cell phone-based data connections that are widely available in low-income countries	Ministry of Health and national tuberculosis programme reporting systems need to be adapted to incorporate test results
	Limited ability to access test results and aggregate these by district, province or national level	Pilot a remote monitoring system for Xpert® that automatically transmits test results to a central database
Determining an appropriate testing algorithm	Balancing the high cost of Xpert® with increased sensitivity for detecting tuberculosis and rifampicin resistance	Start using Xpert® as a first-line test for high risk patients (those with HIV infection, diabetes, cancer, children) and those at risk for MDR-TB
Xpert® durability	Poor understanding of routine maintenance requirements in dusty, non-temperature controlled remote laboratories	Use Xpert® at health centres with adequate staff and maintenance support
		Develop maintenance protocols specific to remote laboratories in Mozambique
Lusophone staff struggling with English software	Software was initially only available in English	A new version was released that supports several languages, including Portuguese

HIV: human immunodeficiency virus; MDR-TB: multi-drug resistant tuberculosis; SMS: short message service.

One computer failed within six months, despite being attached to a surge protector/current stabilizer. Twelve of the original 16 (75%) Xpert® modules broke or failed calibration within 15 months and had to be replaced. In early 2013, Xpert® cartridges and calibration kits were back-ordered, resulting in several days without Xpert® cartridges despite ample lead-time in ordering.^11^ Updating antivirus software and operating systems and transferring data from the local computer to Cepheid in California, USA, was also difficult. Cepheid depends on Xpert® machines being connected to the internet to be able to diagnose problems remotely – this was not feasible in Mozambique and caused significant delays. Over time, shipments of cartridges and customer service improved as Cepheid employees and our team learned how to work with each other. However, importing consumables to Mozambique and clearing customs remained challenging, expensive and time-consuming.

The lack of a notification system to report positive Xpert® results to the national tuberculosis programme and no ability to automatically upload results electronically to a central secure database made it challenging to assess the impact of this intervention. It was difficult to remotely monitor Xpert® testing and notify key individuals in a timely fashion – such as provincial MDR-TB care coordinators when a patient tested positive for rifampicin resistance. WHO guidance on recording and reporting test results should allow national tuberculosis programmes to better monitor performance.^12^

The current two-step testing algorithm likely caused delays in tuberculosis diagnosis relative to using Xpert® as a first-line test. Some patients returned after 24 hours for their smear results, but did not get their subsequent Xpert® result. The need for repeat visits to establish a diagnosis may have led to higher pretreatment loss to follow-up. In the beginning, sites did not routinely collect patient contact information so it was impossible to reach these primary defaulters. Initially there was no protocol to routinely reconcile laboratory and treatment registries to ensure that all patients diagnosed with tuberculosis were started on treatment. Efforts are ongoing to improve the entire tuberculosis care cascade and to increase treatment initiation rates.

## Lessons learnt

Our small-scale implementation project allowed us to gain important practical experience about the strengths and limitations of this new diagnostic technique (Box 1). Rapid tests have the potential to improve the diagnosis of tuberculosis and identify drug resistance. However, the tools are expensive and we experienced problems with the machines, data transfer, linkage to tuberculosis treatment initiation, reporting and follow-up. If the use of rapid diagnostic tests for tuberculosis is expanded to the national level, system improvements are needed to prevent these problems from occurring at a larger scale.

Box 1 **Summary of main lessons learnt**

- Xpert® MTB/RIF (Xpert®) testing increased the number of confirmed bacteriologically positive tuberculosis cases. However, one third of patients diagnosed by Xpert® did not receive tuberculosis treatment, highlighting the need to strengthen the overall tuberculosis care cascade.
- Implementation and maintenance of Xpert® machines are costly and logistically challenging, which creates a need for more affordable and durable molecular testing platforms.
- Of the tuberculosis patients diagnosed with Xpert®, 5% were resistant to rifampicin, highlighting the need to develop a multi-drug resistance tuberculosis programme in Mozambique.

The national tuberculosis programme is committed to effective care of all tuberculosis patients in Mozambique. Improving diagnosis is a part of this commitment and, therefore, rapid tests like Xpert® will be provided to more health centres. However, affordable and durable tuberculosis diagnostic platforms that can handle the stresses of laboratory environments in low-resource settings are still needed. Remote monitoring and evaluation of rapid testing systems may improve with the use of platforms such as GxAlert, XpertSMS and the Cepheid cloud system.^13^ Finally, while innovation in diagnostic tests is important, this is only one part of the tuberculosis care cascade. When increasing the number of people diagnosed with tuberculosis, attention is needed to ensure they receive the appropriate treatment, care and support.
